# Review to Understand the Crosstalk between Immunotherapy and Tumor Metabolism

**DOI:** 10.3390/molecules28020862

**Published:** 2023-01-15

**Authors:** Pratibha Pandey, Fahad Khan, Tarun Kumar Upadhyay, Ramish Maqsood

**Affiliations:** 1 Department of Biotechnology, Noida Institute of Engineering and Technology, Greater Noida 201306, India; 2 Department of Biotechnology, Parul Institute of Applied Sciences and Centre of Research for Development, Parul University, Vadodara 391760, India

**Keywords:** tumor metabolism, immune checkpoint, tumor microenvironment, immunotherapy, tumor immunity

## Abstract

Immune checkpoint inhibitors have ushered in a new era of cancer treatment by increasing the likelihood of long-term survival for patients with metastatic disease and by introducing fresh therapeutic indications in cases where the disease is still in its early stages. Immune checkpoint inhibitors that target the proteins cytotoxic T-lymphocyte-associated antigen-4 (CTLA-4) or programmed death-1/programmed death ligand-1 have significantly improved overall survival in patients with certain cancers and are expected to help patients achieve complete long-lasting remissions and cures. Some patients who receive immune checkpoint inhibitors, however, either experience therapeutic failure or eventually develop immunotherapy resistance. Such individuals are common, which necessitates a deeper understanding of how cancer progresses, particularly with regard to nutritional regulation in the tumor microenvironment (TME), which comprises metabolic cross-talk between metabolites and tumor cells as well as intracellular metabolism in immune and cancer cells. Combination of immunotherapy with targeted metabolic regulation might be a focus of future cancer research despite a lack of existing clinical evidence. Here, we reviewed the significance of the tumor microenvironment and discussed the most significant immunological checkpoints that have recently been identified. In addition, metabolic regulation of tumor immunity and immunological checkpoints in the TME, including glycolysis, amino acid metabolism, lipid metabolism, and other metabolic pathways were also incorporated to discuss the possible metabolism-based treatment methods being researched in preclinical and clinical settings. This review will contribute to the identification of a relationship or crosstalk between tumor metabolism and immunotherapy, which will shed significant light on cancer treatment and cancer research.

## 1. Introduction

Cancer arises from the accumulation of genomic abnormalities in precancerous cells. These cells expropriate key homeostatic functions to promote survival and growth and evade clearance by the immune system. It has been suggested that the interaction between malignant cells and the immune system during cancer development involves three steps: elimination, a subsequent equilibrium step, and escape from immune control [1]. Immune checkpoint inhibitors (CTLA-4, PD-1/PD-L1 based ICIs) have reformed cancer therapeutics by enhancing the immune response against tumor cells. ICIs are now thought to be more effective than conventional treatment regimens. Additionally, due to the specificity of immunotherapy, it may have fewer adverse effects and reduce the risk of developing cancer [2]. Immunotherapy that targets immunological checkpoints is therefore anticipated to help cancer patients by obtaining complete, long-lasting remissions and cures. The majority of patients, however, do not respond well to immunotherapy or inevitably acquire resistance to it after receiving treatment [3]. Immunotherapy resistance is caused by a variety of mechanisms, such as the expression of tumor antigens, antigen presentation, the immunosuppressive milieu, and metabolism or nutrient availability in the tumor microenvironment (TME). In the realm of immunotherapy, these variables lead to several restrictions and difficulties [4]. In this review, we extensively and methodically explore metabolic modulation of tumor immunity and immunological checkpoints in the TME, as well as prospective therapeutic approaches in the treatment of cancer.

The therapeutic options for the treatment of cancer have significantly changed in recent years with the introduction of immunotherapy. A variety of monoclonal antibody-based immunotherapy techniques known as immune checkpoint blockade (ICB) aim to inhibit the activation of inhibitory receptors. Immune cells express them on their surfaces along with their ligands. These therapies primarily target CTLA-4 and PD-1 or its ligand PD-L1. ICB has received a lot of attention, particularly due to the longevity of responses and its impacts on patients’ overall lifespan. Finding patients who are most likely to respond is a major difficulty [5]. Both tumor-intrinsic and tumor-extrinsic settings, such as the tumor microenvironment, anatomical location, and even food, affect the characteristics and behaviors of tumors. Therefore, the tumor microenvironment and internal super-tumor microenvironment (STME), which exists within the tumor and is caused by intrinsic variables (e.g., intra-tumor heterogeneity) or extrinsic ones (e.g., UV causing skin cancer and everlasting beam radiation during cancer therapy), all directly affect cancer growth. The presence of tumor cells that interact with nearby cells, such as endothelial cells, adipocytes, fibroblasts, lymphocytes, immune cells, and cancer-associated fibroblasts (CAFs), has drawn increased focus to the tumor microenvironment.

Numerous metabolic enzymes and pathways are waiting to be investigated as potential targets for cancer treatment. In this regard, it is likely that the study of metabolism would gain the most from the application of systems biology methods to pinpoint important pathways. Each of these cell types has distinct metabolic properties that influence tumor survival and, in turn, the effectiveness of cancer treatment. One of the most prevalent and significant elements of the tumor microenvironment is CAFs. They can influence the growth of cancer cells via their metabolic pathways in addition to promoting tumor progression by the release of different growth factors, cytokines, chemokines, H_2_O_2_, and the breakdown of the extracellular matrix (ECM). Even in the presence of oxygen, CAFs prefer to switch to anaerobic glycolysis and release lactate to fuel tumor cells. They can also induce complementary metabolic pathways to buffer and recycle byproducts of anaerobic metabolism to maintain tumor growth [6,7].

**Figure 1:**
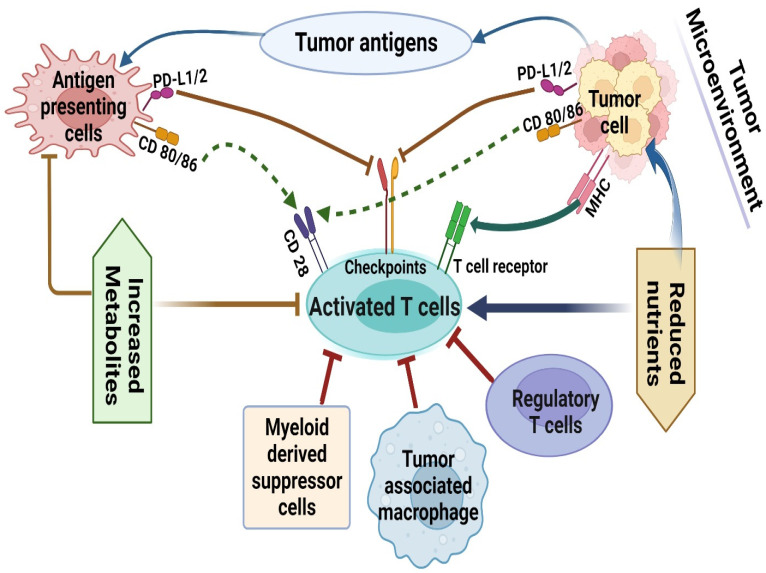
Regulation of immune response by nutrients in a tumor microenvironment. In a tumor microenvironment reduced nutrients and elevated immunosuppressive metabolites modulate the immune response to a tumor. The red line (with bar) denotes inhibition whereas the green dotted line (with arrow) denotes activation.

Additionally, endothelial cells use lactate as a signaling mechanism to encourage angiogenesis and depend more on glycolysis for vessel creation than oxidative phosphorylation. In addition to controlling the movement of other cells into and out of tissues, the endothelium also controls the flow of nutrients, oxygen, and other solutes from the bloodstream to the tissues. Through newly formed vessels, tumors can obtain nutritional support for the growth or transport of cancer cells to other regions of the body. Therefore, original tumor cells are finally enmeshed in a distinct organ-like structure that also contains blood arteries, the ECM, immune cells, stromal cells, and inflammatory cytokines [8,9]. For glucose, amino acids (such as glutamine, arginine, and tryptophan), lipids, proteins, nucleic acids, and other metabolites, tumor cells engage in a complex nutritional competition with other cells, particularly immune cells. Along with other elements (such as hypoxia and an acidic environment), the presence of these nutrients is not only necessary for the development of tumors but also has an impact on immune cell activity [10,11] (Figure 1). Further research into the metabolic control of immune evasion shown in the tumor microenvironment may help create novel therapeutic approaches for immunotherapy resistance. Life-and-death choices are influenced by cellular metabolism. The complicated relationship between metabolic control and cancer growth is an emerging theme in cancer biology. This is partially because the availability of nutrients has strong control over how much an organism can reproduce. Mitogenic signals encourage the intake of nutrients and the production of DNA, RNA, proteins, and lipids. In light of this, it would seem obvious that oncogenes, which frequently stimulate proliferation, would also encourage metabolic alterations. The current state of our knowledge regarding the relationship between “metabolic transformation” and oncogenic transformation is summarized in this article, along with the potential “Achilles’ heel” of cancer—metabolic inhibition. The following subsections would focus more on the potential of cancer and immune cells.

## 2. Immune Checkpoints Regulation

### 2.1. T-Cell Immunoglobulin and Mucin-3 (Tim-3 or HAVCR2) Immune Checkpoint Receptor

Tim-3 is widely expressed in activated T cells, Foxp3^+^ Treg cells, NK cells, and monocytes. A number of immune cells, including leukemic stem cells, express the checkpoint receptor T-cell immunoglobulin and mucin domain 3 (Tim-3). The TIM family of proteins are type I membrane proteins that have a single transmembrane domain, a variable Ig domain (IgV), and a glycosylated mucin domain of different lengths. Except for Tim-4, all TIM molecules have a common tyrosine-based signaling motif in their C-terminal cytoplasmic tail. The Tim-3 checkpoint receptor is an unusual protein that lacks either the typical inhibitory immunoreceptor tyrosine-based inhibition or immunoreceptor tyrosine-based switch signaling motifs in its cytoplasmic tail. The four Tim-3 ligands that have currently been identified are high mobility group protein B1, galectin-9, CEACAM-1, and phosphatidyl serine [12]. Recent research has shown Tim-3 to be a key factor in the exhaustion of CD8+ T cells that occurs in chronic immunological diseases such as cancer and persistent viral infection in both humans and experimental mice. Recent research suggests that Tim-3 can affect cancer outcomes in addition to its role in worn-out T cells by acting on myeloid cells and cancer stem cells. Due to its expression on both defective CD8+ T cells and Tregs—two important immune cell types that contribute to immunosuppression in tumor tissue—the Tim-3 pathway is ideally suited as a target for anticancer immunotherapy. Numerous studies have shown that TIM-3 overexpression may be associated with more aggressive or advanced disease, and poor survival in solid tumors, and may also correlate with these factors. According to certain in vitro investigations, TIM-3 expression levels are linked with the invasion and metastasis of cancer cells. In preclinical models, TIM-3 and PD-1 blockade may slow tumor growth and maybe enhance anti-tumor T-cell responses in cancer patients. It can be used to determine how malignant renal cell carcinoma is. It affects the immune microenvironment and outcome prediction. The TIM-3/Gal-9 pathway induces T-cell exhaustion and is intimately linked to survival, and patients with hepatocellular carcinoma displayed elevated expression of TIM-3 on tissue-associated macrophages and peripheral blood monocytes. Tim-3 expression has been found to be a poor predictive biomarker in a number of tumor types, which is not surprising given its inhibitory effects on a variety of cell types. Insufficient clinical parameters in NSCLC have been linked to the presence of TIM-3^+^ Treg, as was previously discussed [13]. Similar to this, Komohara et al. showed that CD204^+^ tumor-associated macrophages and tumor cells in patients with clear cell renal cell carcinoma expressed TIM-3 strongly and that a higher level of TIM-3 expression was positively correlated with shorter progression-free survival in these patients [14]. According to Li et al., TIM-3 expression was higher on CD4^+^ and CD8^+^ T cells in HBV-associated HCC compared to the surrounding tissues, and the proportion of TIM-3^+^ tumor-infiltrating lymphocytes was inversely related to patient survival [15]. TIM-3 expression has additionally been linked to advanced tumor node metastasis (TNM) stages in a number of other malignancies, including gastric cancer [16], colon cancer [17], and cervical cancer [18]. It is noteworthy that TIM-3 expression levels were strongly connected with overall survival in a meta-analysis of patients with solid malignancies [19].

Tim-3 and PD-1 co-blockade can slow tumor growth in preclinical models and enhance anti-tumor T-cell responses in cancer patients. The four human IgG isotypes (IgG1-4) have different binding affinities for different Fc receptors (FcR) and complement components, including C1q. IgG2 and IgG4 produce substantially lesser or no ADCC and CDC, whereas IgG1 has the strongest affinity to all FcRs and C1q, leading to considerable effector activities, such as ADCC, ADCP, and CDC. The majority of anti-TIM-3 antibodies in early clinical development are Fc-receptor silent, with the exception being Sym023, a wild-type IgG1 antibody that is now being tested in advanced solid tumors and lymphoma (NCT03489343) [20] (Table 1).

Some anti-TIM-3 mAbs use hinge stabilization (S228P) to prevent fab-arm interchange and are hIgG4 isotypes. Recent research has shown that hIgG4 antibodies with the S228P mutation can bind FcRI and mediate ADCP. It needs to be established if clinical anti-TIM-3 antibodies mediate ADCP and if this could be useful in the AML/MDS context where TIM-3 expression on LSCs or blasts may result in direct anticancer activity. Notably, the surrogate anti-TIM-3 mAb that showed effectiveness in inhibiting leukemic engraftment in an immune-deficient murine host was both ADCC-competent and CDC-competent, indicating that optimization of FcR engagement may be a desirable characteristic for anti-TIM-3 mAbs in AML/MDS [21,22].

A combination therapy that entails blocking both the Tim-3 and PD-1 pathways is more successful than blocking only one of the routes. Combination therapy also promotes the growth of tumor antigen-specific CD8^+^ T cells while suppressing Tregs, reversing tumor-induced T-cell exhaustion/dysfunction. Additionally, enhanced anti-tumor immunity was seen in a gastric cancer model with combination therapy that blocked Tim-3, LAG-3, and PD-1, indicating the promise of this approach as a treatment [23,24]. TIM-3 is currently recognized as a negative regulator of anti-tumor immunity. The next generation of immunotherapy should focus on TIM-3 because of a number of its characteristics. For instance, its particular expression on intra-tumoral T cells may enable more precise therapy by targeting tumor-infiltrating T cells, potentially lowering non-specific toxicity. Additionally, TIM-3 signaling differs significantly from those of CTLA-4 and PD-1, which have very well-defined inhibitory pathways. In contrast, depending on the cellular environment, TIM-3 has the ability to both promote and inhibit proximal signaling in T cells [25,26]. Therefore, there is a huge potential for targeting TIM-3 both alone and in combination with current PD-1 and CTLA-4-based immunotherapy of cancer due to the unique expression and intracellular signaling.

### 2.2. Cytotoxic T Lymphocyte-Associated Antigen-4 (CTLA-4) and PD-1 Immune Checkpoint Receptor

Immune checkpoints are co-stimulators or co-inhibitors that closely control T-cell activation. T cells can multiply and move in the direction of a particular antigen when antigen/MHC and TCR (T-cell receptor) binding are combined with the activation of costimulatory receptors, such as CD28. On the other hand, T-cell activation will be suppressed if antigen/MHC and TCR interaction is accompanied by the activation of coinhibitory receptors, such as CTLA-4. Although it is not visible in naive T cells, CTLA-4 is quickly activated upon T-cell activation and principally controls the amplitude of T cells during the initial priming phase in lymphoid organs. Eventually, excessive immunity is thwarted by the binding of CTLA-4 to B7 proteins, which competes with CD28 costimulatory signals. CTLA-4 (also known as CD152) plays a crucial role in the discovery and advancement of immunotherapy as the first checkpoint inhibitor (ipilimumab) authorized by the Food and Drug Administration (FDA). Both the TCR and the B7-CD28/CTLA-4 co-stimulatory signals must be active for T cells to become activated [27]. The CTLA-4 protein is kept in the Golgi in naive T cells, but it is promoted to form a CTLA-4-containing vesicle when it binds to the T-cell receptor interacting molecule (TRIM). When the TCR binds to the major histocompatibility complex (MHC), CTLA-4 dissociates TRIM and moves from the vesicle to the membrane, where it competes with CD28 for trans-endocytosis binding with CD80 or CD86 [28,29]. The clathrin adaptor protein 2 (AP-2) binds to the unphosphorylated CTLA-4 cytoplasmic domain (YVKM motif), which facilitates endosome and lysosome internalization quickly. Upon T-cell activation, AP2 is released from the same tyrosine site as CTLA-4 and phosphorylated at the tyrosine site of the YVKM motif. The beige-like, lipopolysaccharide-responsive anchor protein then regulates the return of CTLA-4-containing endosomes to the membrane (LRBA). CTLA-4 is then transported from the Golgi to the lysosome for destruction by adaptor protein 1 (AP-1) [30]. CTLA-4 has the ability to block T-cell activation, cell growth, and naive CD4+ T-cell differentiation. When effector regulatory T cells (Tregs) are selectively eliminated by the application of a CTLA-4 neutralizing antibody, effector CD8+ T cells that are normally inactive against tumors become active.

The maintenance of peripheral tolerance is greatly aided by PD-1, as opposed to CTLA-4. When PD-1 is activated by its ligands, Src homology 2 (SH2) domain-containing phosphatases 1/2 (SHP1/2) are attracted and subsequently recruited, which inhibits T-cell proliferation and cytokine production that is mediated by TCR [31,32]. Some cancer cells can produce inhibitory ligands that can bind to co-inhibitory receptor molecules. This interaction reduces typical anti-tumor immunological responses, aiding immune escape. Therefore, blocking these immunological checkpoints may activate anti-tumor immune responses in patients. Instead of directly killing cancer cells, therapies involving immune checkpoint blockade reactivate endogenous anti-tumor activity by leveraging the host immune system [33].

The PD-1/PD-L1 pathway is a central mediator of immunosuppression in the tumor microenvironment. PD-1 and PD-L1 or PD-L2 activity regulates T-cell activation, proliferation, and cytotoxic secretion to inhibit anti-tumor immunity in cancer. PD-1 is found on the surface of immune cells and contains an immunoreceptor tyrosine inhibitor conventional motif (ITIM) and an immunoreceptor tyrosine switch motif (ITSM). The binding of the inhibitory phosphatase SHP-2 to these motifs inhibits TCR-mediated immune function and prevents T cells from binding to cognate tumor peptide–MHC complexes within the TME [34,35]. PD-L1, one of the PD-1 ligands, is widely overexpressed on tumor cells and infiltrating leukocytes, in contrast to the ligand of CTLA-4. As a result, tumor cells have the ability to cause PD-1-mediated T-cell exhaustion, and blocking either PD-1 or PD-L1 results in increased anti-tumor cytotoxic T-cell responses. The fact that PD-1- and PD-L1-defective mice exhibit relatively modest symptoms and later onset of organ inflammation emphasizes the importance of the PD-1 pathway in tumor treatment [36]. In comparison to the monoclonal antibody against CTLA-4, blocking PD-1 or PD-L1 produces less severe and frequent autoimmune side effects [37]. Notably, PD-L1 promotes Foxp3 expression, causes the conversion of naive CD4+ T cells to Tregs, and activates the immunosuppressive activity of Tregs to promote the formation and function of Tregs. PD-1 and PD-L1 control the growth of Tregs through the Notch pathway. Patients with immunological thrombocytopenic purpura who have PD-1/PD-L1 activated have an unbalanced ratio of Th1/Th2 and Treg/Th17 cells. Additionally, PD-1 inhibition encourages PD-1 effector Treg cell growth, which suppresses anti-tumor immunity [38,39].

### 2.3. Lymphocyte Activation Gene 3 (LAG-3) Immune Checkpoint Receptor

Triebel and colleagues first identified LAG-3 (CD223) in 1990. The LAG-3 gene has 8 exons, and the corresponding cDNA can code for a type I membrane protein with 498 amino acids. On chromosome 12, the LAG-3 gene lies next to the CD4 gene, and subsequent examination of the amino acid sequence reveals that the two genes are about 20% identical [40,41]. The mature LAG-3 protein is composed of four regions: the cytoplasm, the transmembrane region, the extracellular area, and the hydrophobic leader. Four domains that are similar to the immunoglobulin (Ig) superfamily make up the extracellular area (D1-D4). A distinctive short amino acid sequence known as the “extra loop” is present in the membrane-distal D1 domain [42]. A serine phosphorylation site, a KIEELE motif, and glutamic acid–proline repeats are all conserved areas in the cytoplasmic domain of LAG-3, with the KIEELE motif being necessary for LAG-3 to have an inhibitory effect. A soluble LAG-3 (sLAG-3) is produced when metalloproteases cleave LAG-3 within the linking peptide between the D4 transmembrane domain and the transmembrane domain. Some studies showed that sLAG-3 could reduce the intensity of T-cell immunological responses [43]. In addition to plasmacytoid dendritic cells (pDCs), LAG-3 is commonly expressed on activated CD4+ and CD8+ T cells, Tregs, a subpopulation of natural killer (NK) cells, B cells, and NK cells generally. The activation, proliferation, and secretion of cytokines by T helper 1 (Th1) cells are all negatively regulated by LAG-3 signaling, according to a large body of evidence. These pathways are used by tumor cells to evade immune monitoring during carcinogenesis and the spread of cancer [44,45]. MHC-II is logically regarded as a ligand for LAG-3 given the structural similarity between LAG-3 and CD4. In contrast to CD4, LAG-3 and MHC-II have a 100-fold higher binding affinity. Galectin-3, LSECtin, a-synuclein, MHC-II, and other proteins have now been characterised as interacting with LAG-3, with MHC-II serving as the standard ligand [46].

Since LAG-3 plays a crucial part in immunological control, it has been shown that abnormal LAG-3 expression is associated with a number of illnesses, including cancer, persistent viral infection, parasitic infection, and autoimmune. A translational use of the synergistic approach between LAG-3 and PD-1 in cancer is highlighted by the positive clinical effects of LAG-3 blockage given the immune regulatory function of LAG-3. LAG-3 and PD-1 work together to suppress T-cell signaling and speed up the trafficking of the immunological synapse. Multiple established cancers that are resistant to PD-1 blockade alone are cleared as a result of the dual blockade of LAG-3 and PD-1 [47,48]. Another study confirmed that LAG-3 is significantly upregulated when PD-1 is inhibited and that blocking both checkpoints increases IFN production, highlighting the importance of LAG-3. Consequently, a prospective therapy for malignancies is the synergic strategy combining LAG-3 and PD-1 blockage to overcome the resistance of PD-1 blocking [49].

## 3. Metabolic Regulation of Tumor Immunity

The heterogeneity of cancer as a disease is now recognized by academics and medical professionals working in domains relevant to cancer. Even within the same tumor, distinct cancer cells can exhibit vast biological differences, as can patients with the same type of cancer. Cancer metabolism also demonstrates significant variety, with tumors adopting diverse metabolic pathways that best suit a given microenvironment. However, a considerable number of cancers share certain characteristics with one another regarding cancer metabolism. Alterations in the metabolism of glucose, glutamine, and mitochondria are among these typical characteristics of cancer. These shared characteristics might make it possible to create new therapies that focus on a fundamental aspect of cancer biology. Given that it affects how nutrients are distributed in the body, how immune cells function, and how cancer treatment is affected, tumor metabolism is becoming more and more significant. After immunological checkpoint-based immunotherapy, metabolic control of tumor immunity is emerging as a new area of scientific interest in the fight against cancer.

### 3.1. Combinatorial Therapeutic Approach via Targeting Glycolysis and Immune Checkpoint Inhibitors

The most significant components of the adaptive immune system, T cells, are essential for the effective and focused host defense against cancers. Most cancer cells multiply and survive best in an aerobic glycolysis environment, as opposed to T cells, which rely on the oxidative phosphorylation pathway (also known as the Warburg effect). However, aerobic glycolysis is required for T-cell activation, activity, and differentiation [50,51]. Increased glucose absorption has been one of the most prominent characteristics of malignant tumors. During depletion of glucose levels, immune cells are not able to attack cancer cells, thereby allowing cancer cells to utilize a significant amount of glucose from the environment in the tumor microenvironment for their maximal growth and proliferation. Low-sugar environments can also cause metabolic reprogramming, which can cause tumor cells and TILs to compete with one another for energy, promoting carcinogenesis. Through increased N-glycosylation and the EGFR/ERK/c-Jun pathway, tumors sustain the expression of PD-L1 to regulate glucose metabolism. PKM2, which turns phosphoenolpyruvate into pyruvate, is widely expressed in tumors and aids in the development of malignancies. Unexpectedly, PKM2 and HIF-1 directly bind to the hypoxia response element sites on the PD-L1 promoter, increasing the expression of PD-L1 in both immune and tumor cells. Contrastingly, mTOR activity in T cells is inhibited by glucose competition when PD-L1 and B7-H3 expression is found in tumor cells, where it promotes aerobic glycolysis by activating the PI3K-Akt-mTOR pathway. Anti-PD-L1 and anti-CTLA-4 antibodies can also raise T-cell activity and extracellular glucose levels in the TME [52,53,54]. Additionally, anti-PD-L1 and anti-CTLA-4 can raise T-cell glycolytic activity and restore extracellular glucose levels in the TME. The Akt pathway is less phosphorylated and activated due to the CTLA-4 route, which negatively affects T-cell glucose metabolism and mitochondrial remodeling. CD28-mediated co-stimulation is competitively inhibited by the CTLA-4 pathway. Therefore, the metabolism of the tumor and immune cells is controlled by the interaction between immunological checkpoints and their ligands, such as PD-1/PD-L1 and CTLA-4/CD86 [55].

During T-cell activation, leucine or glucose metabolism inhibition results in an incompetent phenotype. Numerous studies have shown that metabolic reprogramming is one of the mechanisms by which immunological checkpoints in immune cells function. In part by resuming glycolysis and beneficial anabolic processes, immune checkpoint blockage of these pathways recovers the effector activity of TILs. The limitation of glucose caused by tumors is reversed by antibodies against CTLA-4, PD-1, and PD-L1, which also restore IFN-production and glycolysis in T cells. The effect of PD-1 on the metabolism of myeloid cells is yet unknown, however targeting PD-1 that is unique to myeloid cells inhibits tumor growth more effectively than targeting PD-1 that is exclusive to T cells [56,57].

Tumor cells use more aerobic glycolysis than normal cells when meeting their growth-related needs. However, research on inhibiting tumor glycolysis in conjunction with ICIs is lacking, most likely because immune cell activation also depends on glycolysis. For instance, in the mouse B16 melanoma model, phosphofructokinase 2/ fructose-2, 6-bisphosphatase 3 (PFKFB3) stimulates glycolytic activity and lactic acid generation in tumor cells, while PFK-158, its inhibitor, enhances the therapeutic response of antibodies against CTLA-4 [58,59]. Therefore, lowering the glycolytic metabolism of cancer cells may successfully decrease cancer cell proliferation, but it may also inhibit the growth and functionality of immune effector cells that infiltrate tumors.

According to the most recent research, rational combination immunotherapy approaches that include inhibitors of the hypoxia-CD39-CD73-A2aR pathway hold significant promise for enhancing clinical results (Table 2). Targeting adenosine pathways in combination with various ICIs has been the subject of several clinical trials, including the combination of CD73 antibody with chemotherapeutic medicines and ICIs (NCT03616886) and CD73 antibody with stereotactic body radiotherapy and ICIs (NCT03875573; NCT03875573). Additionally, individuals with PD-L1-positive NSCLC are also being tested with adenosine receptor inhibitors, PD-1 inhibitors, and TIGIT inhibitors (NCT04791839, NCT04262856). 

### 3.2. Combinatorial Therapeutic Approach via Targeting Amino Acids and Immune Checkpoint Inhibitors

Numerous malignancies have an abnormal overexpression of amino acid metabolism, more specifically, a dependence on a particular amino acid. The development of effector T lymphocytes depends on the metabolism of glutamine, one of the most typical catabolic amino acids. In addition to impairing T-cell activation and function, glutamine deficiency changes the expression of genes that control glutamine metabolism in DCs. The TCA cycle is powered by glutamine, which is broken down into glutamate and ammonia and then transformed into α-ketoglutaric acid (-KG). Additionally, uridine diphosphate-acetylglucosamine (UDP-GlcNAC), which keeps c-myc expression and the glycosylation of proteins as a substrate, is created. Additionally, glutaminolysis and the availability of amino acids affect the expression of c-myc and HIF-1, which are both stimulated by mTOR [60,61].

The production of other nutrients, including lipids, amino acids, and nucleic acids, can also be boosted by c-myc, including aerobic glycolysis, glutaminolysis, and glycolysis. C-myc protein is exclusively expressed in lymphocytes with high rates of protein synthesis and amino acid uptake due to its short half-life. These elements interact to support the metabolic phenotype of effector T cells. In other words, effector T-cell differentiation can be regulated by altering these pathways. Overexpression of inhibitory receptors, such as CTLA-4 or PD-1, also prevents the increase of glucose and glutamine metabolism after TCR involvement and co-stimulation, which results in worn-out T cells [62,63]. Additionally, glutamate controls the development of cytokines and T-cell proliferation. Both CD4+ and CD8+ T cells upregulate glutamate receptors and boost IFN- production following T-cell activation caused by the interaction of the MHC from the APC with the TCR from the T cells [64]. Additionally, the differentiation of macrophages to control immune responses is aided by the production of α-ketoglutarate (KG) by glutaminolysis.

T-cell activation and immune response control are significantly influenced by arginine metabolism. The oxidation of arginine by the enzyme nitric oxide synthase (NOS) to citrulline and nitric oxide (NO) or the hydrolysis of arginine by the enzyme arginase (ARG1 or ARG2) to ornithine and urea results in the loss of arginine, which may prevent T-cell proliferation and cause immunological escape. By switching activated T cells’ metabolism from glycolysis to oxidative phosphorylation, high intracellular levels of L-arginine can increase T-cell survival [65,66]. On the other hand, arginine deprivation prevents T cells from entering the G0-G1 phase, suppresses cell growth, and reduces their functionality. ARG1 is expressed by MDSCs and activated TAMs, which worsens the consumption of arginine and fosters a milieu harmful to T-cell survival, resulting in tumor immunosuppression [67,68]. High plasma ARG2 concentrations can also increase the microenvironment’s inhibitory effect by preventing T cells and hematopoietic progenitor cells from proliferating [69]. Immunoregulatory cells that express the ARG1 protein in the TME, such as M2-like TAMs, tolerogenic DCs, and Treg cells, prevent T cells from accessing arginine, which inhibits anti-tumor immunity. Acidosis has been shown to increase ARG activity and induce high levels of induced NOS (iNOS) in a number of cancer types [70]. Additionally, depending on NO concentration, exposure duration, and NO sensitivity, the NO generated by these enzymes can either promote or prevent tumor progression and metastasis. T cells may undergo apoptosis when exposed to peroxynitrite, a reactive nitrogen species (RNS) created by the reaction of reactive oxygen and NO. The activation, growth, and effector function of T cells are adversely affected by the buildup of RNS in the TME [71,72]. ARG inhibitors are now being investigated for tumor immunotherapy, examples of which include CB-1158 and 6-gingerol. Additionally, in vitro arginine supplementation promoted the cytotoxicity of T and NK cells, and the generation of effector cytokines, in conjunction with anti-PD-L1 antibody, greatly improved the anti-tumor immune response and prolonged the survival of osteosarcoma mice. The development of central memory-like T cells with enhanced anti-tumor activity is encouraged by arginine supplementation during T cell in vitro growth [73,74,75,76,77]. In order to reactivate T cell- and NK cell-mediated immune responses, arginine supplementation and blockade of arginine degradation in the TME are crucial.

The anti-tumor immune system is impacted by tryptophan metabolism, which is involved in the de novo creation of nicotinamide adenine dinucleotide (NAD+). Both tumor and immune cells in the TME express and release indoleamine 2, 3-dioxygenase (IDO), an enzyme that breaks down tryptophan at a rate that is rate-limiting [78,79]. Tryptophan is broken down by IDO into kynurenine (Kyn), which causes a local tryptophan shortage and subsequently inhibits T cells. Loss of tryptophan causes a rise in uncharged transfer RNA (tRNA), which triggers a GCN2-mediated all-encompassing stress response. A GCN2 direct sensor is triggered when cells have restricted access to amino acids, which stimulates free radical reprogramming of cell function, immunological cell-cycle arrest, and autophagy. Consuming tryptophan can also directly activate GCN2, encourage Treg differentiation, and impair T-cell activity [80,81].

Cancer treatment is actively pursuing targets for amino acid metabolism. In order to reactivate T cell- and NK cell-mediated immunological responses, it is appealing to supply arginine in the TME or to inhibit arginine breakdown. These approaches are now being investigated in clinical studies, such as the combination of the Arg1 inhibitor CB-1158 with the ICI pembrolizumab monoclonal antibody and the combination of the arginine-eliminating ADI-PEG 20 with ICIs to treat solid tumors. Second, tryptophan catabolism can be halted by utilizing IDO1 or TDO inhibitors as a potential cancer treatment. Epacadostat, indoximod, and navoximod are examples of IDO1 inhibitors whose use alone has demonstrated inadequate efficacy in clinical trials. As a result, immune checkpoint inhibitors have been used in combination therapy in current IDO1 inhibitor studies. Epacadostat with the PD-1 inhibitor pembrolizumab showed extensive anti-tumor effectiveness against several cancer types in the ECHO-202/KEYNOTE-037 trial [82]. However, the ECHO-301/KEYNOTE-252 study of epacadostat + pembrolizumab for the treatment of metastatic or unresectable melanoma failed to demonstrate an increase in progression-free survival over pembrolizumab + placebo [83]. In phase I-III clinical trials, epacadostat and pembrolizumab are being used to treat patients with advanced cancers. One such trial is using epacadostat and pembrolizumab along with electroporation therapy to treat head and neck squamous cell carcinoma. Currently, nivolumab, an anti-PD-1 antibody, and ipilimumab, an anti-CTLA-4 antibody, are being combined in clinical trials to assess the efficacy of linrodostat (BMS-986205). Additionally, the oncolytic virus is being evaluated for the treatment of patients with non-muscle invasive bladder cancer in combination with IDO and PD-1 inhibitors. Finally, CB-839 increases the infiltration of effector T cells in tumors and amplifies the anti-tumor effects of these checkpoint inhibitors in mice melanoma models with high mutation burden tumors [84].

### 3.3. Combinatorial Therapeutic Approach via Targeting Lipids and Immune Checkpoint Inhibitors

An elevated rate of lipogenesis is another characteristic of cancer cells. The newly created lipids will be used to create membranes, specifically lipid rafts and signaling molecules with lipid modifications. A possible target for anti-tumor therapy is lipid metabolism, which is becoming more widely recognized. The success of cancer therapy by focusing on lipid metabolism is directly influenced by the way immune cells behave in the tumor microenvironment. Lauric acid, for instance, is a long-chain fatty acid that can boost the differentiation of pro-inflammatory Th1 and Th17 cells and accelerate LPS-induced DC maturation dependent on TLR4 and T-cell activation, while propionic acid, a short-chain fatty acid, can encourage the growth of Treg cells. Furthermore, it has been shown that the anti-CTLA-4 activity in cancer is constrained by short-chain fatty acids. Myeloid cells from the bone marrow become immunosuppressive M2-like TAMs when exposed to oleic acid. Both polyunsaturated and saturated fatty acids work as TLR4 agonists, which can activate NF-kB and JNK, increase the production of TNF-a, and encourage DC maturation [85,86,87,88,89,90,91]. Docosahexaenoic acid (DHA) reduces DC maturation, but eicosapentaenoic and arachidonic acids can also influence T-cell responses, MODC differentiation, and cytokine production. The TLR4 signaling pathway can be disrupted in mature DCs by high-density lipoprotein (HDL) and low-density lipoprotein (LDL). Mature DCs favor glycolysis, whereas tolerogenic DCs depend on OXPHOS and FAO under the control of PPAR and vitamin D3. DC dysfunction can result from abnormal lipid buildup, which can also downregulate CD86, a co-stimulatory molecule, and overexpress IL-10, an anti-inflammatory cytokine [92].

Several lipogenic enzymes, such as fatty acid synthase (FAS), acetyl-CoA carboxylase (ACC), and ATP citrate lyase, have been investigated as potential targets in anti-neoplastic therapy (ACL). Tumor growth is inhibited by their downregulation through pharmacological inhibition or siRNA expression. Fatty acid synthase (FASN) inhibition can partially restore the function of tumor-infiltrating DCs. Tofa, an acetyl-CoA carboxylase (ACC) inhibitor, can block lipid synthesis and restore lipid levels in DCs, restoring their activity and greatly enhancing the effectiveness of cancer vaccines [93,94]. The anti-tumor activity of CD8+ T lymphocytes has been shown to be regulated by cholesterol metabolism. ACAT1 inhibition boosts CD8+ cell proliferation and effector activity while elevating plasma membrane cholesterol levels. The anti-tumor effectiveness can be increased by combining ACAT1 inhibitor and anti-PD-1 antibody therapy [95]. Avasimibe, a sterol O-acyltransferase 1 inhibitor, has also been utilized to prevent cholesterol esterification, which raises cholesterol levels and impairs T-cell effector function and proliferation, improving the anti-tumor impact [96]. Targeting lipid metabolism in cancer is still in the early stages of clinical use. However, numerous preclinical investigations have produced encouraging outcomes. For instance, it has been discovered that the ACAT inhibitor avasimibe, the COX2 inhibitor celecoxib, and the specific EP4 antagonist E7046, when combined with PD-1 or CTLA-4 inhibitors, generate a synergistic anti-tumor immune response in mice tumor models [97,98]. The results taken together imply that lipid synthesis has a significant impact on immune cells, but further research is still required to determine how lipid synthesis affects tumor immunity.

### 3.4. Therapeutic Approaches Involving Combination of Immune Checkpoint Inhibitors and Other Traditional Treatments

In addition to being paired with immune checkpoint inhibitors and metabolic medicines, ICIs have also been used in combination with conventional cancer therapies such as surgery, radiation therapy, chemotherapy, and targeted therapy. Immune checkpoint inhibitors are now being tested for use in the neoadjuvant (preoperative) treatment phase, however, surgery is still the most efficient treatment option for the majority of solid tumors. Neoadjuvant immunotherapy may offer more pronounced benefits than standard neoadjuvant therapy, which enables the tumor to be completely excised and enhances local disease control when the tumor stage is decreased with chemotherapy or radiotherapy [99,100,101,102]. For instance, antigen exposure will dramatically increase the intensity and persistence of tumor-specific T-cell responses. Neoadjuvant immunotherapy can also remove tumor micrometastases in naturally occurring metastatic cancer and lower the likelihood of distant recurrence, extending survival in treatable diseases [103,104,105]. Clinical research has demonstrated the viability of ICIs as a neoadjuvant therapy for melanoma, NSCLC, breast cancer, and liver cancer [106,107,108,109].

Chemotherapy is believed to have a positive immunomodulatory effect and enhance the anti-tumor immune response by increasing antigen expression on the surface of tumor cells, inducing immunogenic cell death of tumor cells, altering immune cell subsets, enhancing effector lymphocytes or lowering inhibitory immune cells [110,111,112]. Atezolizumab combination with carboplatin + albumin paclitaxel + atezolizumab exhibits higher anti-tumor immune response in patients with stage IV non-squamous NSCLC as compared to chemotherapy alone. As a first-line treatment for both non-squamous and squamous NSCLC, pembrolizumab in combination with chemotherapy was found to be more successful [113,114,115].

Chemotherapy can cause lymphopenia and bone marrow suppression, even though it can be used with immunotherapy to have superior therapeutic benefits. Additionally, pretreatment with several chemotherapy medications additionally employs glucocorticoids, which will heighten immunosuppression. Additionally, after receiving short-term ipilimumab treatment, patients with melanoma received radiotherapy for the paraspinal lesions. The results revealed that the perihilar lymph nodes and splenic metastases that were not exposed to radiation also reduced over time [116]. Additionally, it was discovered that radiotherapy paired with dual immune checkpoint blockers had a better outcome for patients with malignant melanoma than radiotherapy combined with CTLA-4 blockers. Anti-angiogenic therapy can encourage the normalization of tumor blood vessels and facilitate the invasion of tumor CD8+ T cells. For instance, pretreatment with bevacizumab increased the impact of immunotherapy medications in patients with colorectal cancer due to killer T cells entering the TME after the normalization of blood vessels [117,118,119]. Altogether, this review clearly indicates that immune checkpoint inhibitors, combined with targeted drugs, can be used as the first-line treatment of unresectable carcinomas.

## 4. Conclusions

The metabolic control of tumor immunity is characterized by several key characteristics that have an impact on immunological checkpoint-based immunotherapy. The most significant factor, which has a direct impact on both the immune and clinical responses of cancer patients, is the metabolic battle for scarce nutrients between immune and cancer cells. Tumors can compete for nutrients (such as glucose, amino acids, and lipids) from the TME to meet their requirements for growth by developing specific abilities through adaptation to a diverse and dynamic microenvironment. Competition for these resources causes a shortage of these nutrients, which in turn inhibits immune cell function, particularly T-cell growth and effector functions, which ultimately results in T-cell exhaustion and lowered immunological responses against tumors. Response to immune checkpoint-based immunotherapy is influenced by the interaction between metabolic reprogramming and immunological checkpoints. Immune checkpoint expression and stability are significantly increased by nutrient metabolism, especially glycolysis and lipid metabolism, in cancer cells. In addition, the metabolism of immune cells is impacted by both immunological checkpoints and costimulatory signals. These interactions influence the growth and activation of T cells and impact the outcome of immune checkpoint blockade therapy. Tumor microenvironment looks to be a complicated tumor ecosystem due to metabolic interaction between the various cell types. The TME also contains immune cells, endothelial cells, fibroblasts, lymphocytes, and CAFs in addition to tumor cells. In addition to their direct or indirect interactions with tumor cells, these cells also secrete or create other substances that have an impact on the ecosystem surrounding the tumor, such as metabolites, growth factors, cytokines, chemokines, and ECM. The most crucial task for the advancement of tumor immunotherapy is to uncover intricate regulations. It is evident that altering the tumor ecology or understanding the differences between cancer and immunologic metabolic reprogramming would open up chances to eradicate tumors by improving the effectiveness of immunotherapy. It is critical to comprehend how a tumor operates if it is thought of as a form of organ. For instance, how does it protect itself from immune cell attack and how does it receive external nutrients? These aspects affect whether the tumor can survive in vivo. Therefore, understanding the function of organ-like tumors requires knowledge of tumor immune escape mechanisms and metabolic interaction in the TME. It is still unclear how specific regulation mechanisms for immune cells work, in addition to how nutrients affect them. Thus, identification of new metabolic regulators will enhance immunotherapy by providing new targets. With more research into tumor metabolism, immunotherapy failure or resistance may be diminished or even eliminated.

## Figures and Tables

**Table 1:** Clinical trials initiated with Tim-3 antibodies.

Clinical Trials.gov Identifier	Reagent Name	Co-Blockade	Cancer	Manufacturer	Year
NCT02608268	MGB453	Anti PD-1	Patients with advanced cancer	Novartis Pharmaceuticals	2015
NCT02817633	TSR-022	Anti PD-1	Patients with advanced solid tumors	Tesaro, Inc.	2016
NCT03066648	MGB453	Monotherapy/anti-PD-1/Hypomethylating agent	MDS/AML	Novartis Pharmaceuticals	2017
NCT030680508	TSR-022	Anti PD-1	Liver cancer	Tesaro, Inc.	2016
NCT03099109	LY3321367	Anti PD-L1	Advanced solid tumors (relapsed/refractory)	Eli Lilly and Company	2019
NCT03311412 NCT03489343	Sym023	Anti PD-1/Monotherapy	Lymphomas and solid tumors	Symphogen	2018
NCT03708328	R07121661	TIM-3 and PD-1	Solid tumors metastatic melanoma	Hoffmann-La Roche	2019
NCT03744468	BGBA425	Anti PD-1	Solid tumors	BeiGene	2017
NCT03946670	MGB453	MDS	Randomized/HMA	Novartis Pharmaceuticals	2019

**Table 2:** Clinical trials using immune checkpoint inhibitors for metabolic treatments.

Clinical Trial	Drugs	Cancer	ICIs	Targets	Mechanism
NCT03684811	Olutasidenib (FT-2102)	Hepatobiliary tumors	nivolumab (PD-1)	TCA Cycle	Inhibits the tumor growth and oncometabolite 2-HG production
NCT04056910	Ivosidenib (AG-120)	Gliomas, advanced solid tumors	nivolumab (PD-1)		
NCT03048500	Metformin	Non-small-cell lung cancer	nivolumab (PD-1)	Oxidative phosphorylation	Inhibition of ATP synthesis and tumor growth, AMPK activation
NCT03800602		Colorectal cancer	nivolumab (PD-1)		
NCT03311308		Melanoma	pembrolizumab (PD-1)		
NCT04114136		Solid tumors	nivolumab (PD-1) pembrolizumab (PD-1)		
NCT03994744		Small-cell lung cancer	sintilimab (PD-1)		
NCT04414540		head and neck squamous cell carcinoma	pembrolizumab (PD-1)		
NCT03618654		head and neck squamous cell carcinoma	durvalumab (PD-L1)		
NCT02903914	CB-1158	Advanced solid tumors	pembrolizumab (PD-1)	Arginine	Inhibition of arginine degradation
NCT03254732	ADI-PEG20	Advanced solid tumors	pembrolizumab (PD-1)		Promotes degradation of tumor growth promoting arginine
NCT03922880		Uveal melanoma	nivolumab (PD-1) + ipilimumab (CTLA-4)		
NCT04899921	Trigriluzole (BHV-4157)	lymphoma Solid tumors	ipilimumab (CTLA-4) + nivolumab (PD-1)	Glutamine	Reduction in extracellular level via inhibition of the release of T cells
NCT03229278		Renal cell carcinoma, melanoma, Non-small-cell lung cancer	nivolumab (PD-1) or pembrolizumab (PD-1)		
NCT02771626	CB-839	Renal cell carcinoma, melanoma, Non-small-cell lung cancer	nivolumab (PD-1)		Inhibition of cancer cell proliferation and glutaminolysis
NCT03361865	Epacadostat	Urothelial cancer	pembrolizumab (PD-1)	Tryptophan	Inhibition of Trp-Kyn-AhR pathway and upregulation of tumor immunity
NCT03322540		Non-small-cell lung cancer			
NCT03374488		Urothelial cancer			
NCT03291054		Gastrointestinal stromal tumors			
NCT03260894		Renal cell carcinoma			
NCT02364076		Thymic carcinoma			
NCT03358472		head and neck squamous cell carcinoma			
NCT03414229		Sarcoma			
NCT02364076		Thymic carcinoma			
NCT03196232		Gastric cancer			
NCT02298153		Non-small-cell lung cancer, Urothelial cancer			
NCT02752074		Melanoma			
NCT03463161		Head and neck cancer			
NCT03348904		Non-small-cell lung cancer			
NCT03602586		Ovarian cancer			
NCT03823131		head and neck squamous cell carcinoma	pembrolizumab (PD-1) + EPT		
NCT02178722		solid tumors	pembrolizumab (PD-1)		
NCT02318277		Advanced solid tumors	durvalumab (PD-L1)		
NCT01604889		Melanoma	ipilimumab (CTLA-4)		
NCT03707457	Linrodostat (BMS-986205)	Glioblastoma	nivolumab (PD-1)		
NCT03695250		Hepatocellular carcinoma			
NCT03854032		HNSCC			
NCT04106414		Endometrial cancer			
NCT03329846		Melanoma			
NCT02996110		RCC			
NCT03192943		Advanced tumors			
NCT02935634		Gastric cancer			
NCT03335540		Advanced solid tumors			

## Data Availability

Not applicable.
